# Reverse sural fasciocutaneous flap with a cutaneous pedicle to cover distal lower limb soft tissue defects: experience of 109 clinical cases

**DOI:** 10.1007/s10195-014-0304-0

**Published:** 2014-06-24

**Authors:** Anoop C. Dhamangaonkar, Hemant S. Patankar

**Affiliations:** Patankar’s Hand and Limb Reconstruction Clinic, 204, Garodia Market, Plot no. 8-A, D.K. Sandu Marg, Chembur, Mumbai, 400071 Maharashtra India

**Keywords:** Distal, Defects, Flaps, Sural

## Abstract

**Background:**

Soft tissue defects over the mid- and distal third tibia, heel, dorsum and plantar aspect of the foot and over the medial, lateral and posterior aspect of the ankle are a common scenario in clinical orthopaedic practice. In this article, we describe the utility of the reverse sural fasciocutaneous flap with a cutaneous pedicle in 109 clinical cases with distal lower limb soft tissue defects.

**Materials and methods:**

A total of 109 patients were operated on for moderate (5–15 cm) and large (more than 15 cm) soft tissue defects at various sites along the lower limb including foot, heel and sole with the reverse sural fasciocutaneous flap. The defects were secondary to trauma (61 cases), diabetic ulcers (12 cases), post-traumatic scar contracture (8 cases), venous ulcer (4 cases), wound dehiscence (10 cases), leprotic non-healing ulcer (1 case), post-infective wound (1 case), radiation-induced ulcer following radiotherapy for synovial cell sarcoma (1 case), post-fibromatosis excision (1 case), post-dermatofibrosarcoma excision (1 case), post-heel melanoma excision (1 case) and actinomycosis foot (1 case). Patients were assessed for flap uptake and healing of defects.

**Results:**

Among the 102 cases analysed, 81 were male and 21 female with an average age of 32.7 years. The average size of the flaps was 148.10 ± 59.54 cm^2^. The flap healed uneventfully in 89.21 % of patients. Edge necrosis occurred in 9 cases. Donor site regrafting was required in 7 patients.

**Conclusion:**

The reverse sural fasciocutaneous flap with a cutaneous pedicle is a quick, versatile, easy and safe soft tissue defect coverage technique to cover most of the soft tissue defects of the lower limb in common orthopaedic practice and does not require any microvascular repair, though it may be cosmetically unappealing in a few cases.

**Level of evidence:**

IV (Case series)

## Introduction

Soft tissue defects over the distal third of lower limb are a common scenario faced by orthopaedic surgeons in their clinical practice. It may be the result of primary open trauma (Gustilo-Anderson Grade 3B), defect after radical debridement of open fractures (Gustilo Anderson 3A), cellulitis, defects created after contracture release, wound dehiscence after tendo-achilles repair, varicose ulcers and diabetic ulcers. Over the past few decades, many lower limb wound coverage techniques have been described, such as free flaps [1], random local flaps [2], distally based muscle flaps [3, 4], staged and undelayed fascial [5, 6] and fasciocutaneous flaps [7–11] and reverse flow arterial flaps [12, 13]. One such wound coverage technique popularized by Masquelet is the use of neurocutaneous flaps based on distal neurocutaneous perforators or venocutaneous perforators [14]. Since then, reverse sural fasciocutaneous flaps have been described by Hasegawa et al. [15], Rajacic et al. [16] and Nakajima et al. [17], in which the arterial vascularization of the flap is provided by the vascular plexus around and inside the sural cutaneous nerve and by the arterial branches accompanying the short saphenous vein. In this article, we describe the utility of this reverse sural fasciocutaneous flap with a cutaneous pedicle in 109 clinical cases with moderate and large lower limb soft tissue defects.

## Materials and methods

**Fig. 1:**
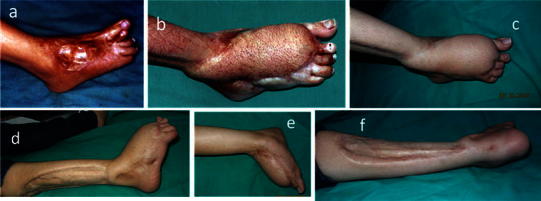
Dorsum of foot soft tissue defect after a post-traumatic scar contracture excision: **a** pre-operative clinical image, **b** 2 years post-operative clinical outcome, **c**–**f** 10 years post-operative clinical outcome

**Fig. 2:**
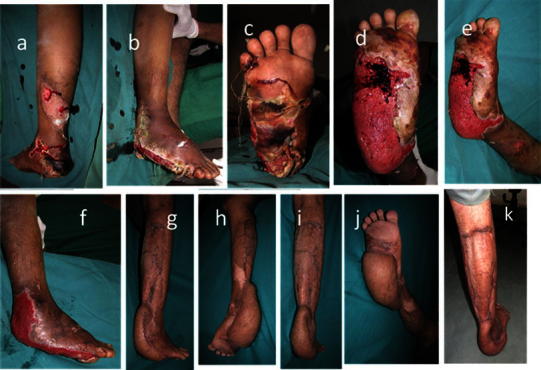
Heel pad avulsion with degloving over plantar foot, heel and rear heel: **a**–**c** clinical image at presentation, **d**–**f** 2 weeks post-presentation, **g**–**j** 1 year post-operative clinical outcome, **k** clinical image of patient standing on the flap at 2 years follow-up

Between 2002 and 2012, 109 adult patients were included in this study. All were operated for moderate (5–15 cm) and large (more than 15 cm) skin and soft tissue defects which were covered by a reverse sural fasciocutaneous flap with a cutaneous pedicle. These defects were over the anterior, medial and lateral ankle; medial, lateral, rear and plantar heel; dorsum (Fig. 1), plantar aspect (Fig. 2) of the foot; anteromedial and anterolateral middle and distal third tibia; over the tendo-achilles; and over an amputation stump. The defect was secondary to trauma (59.80 % cases), diabetic ulcers (11.76 % cases), post-traumatic scar contracture (7.84 % cases), venous ulcer (3.92 % cases), wound dehiscence (9.80 % cases), leprotic non-healing ulcer (0.98 % case), post-infective wound (0.98 % case), radiation-induced ulcer following radiotherapy for synovial cell sarcoma (0.98 % case), post-fibromatosis excision (0.98 % case), post-dermatofibrosarcoma excision (0.98 % case), post-heel melanoma excision (0.98 % case) and actinomycosis foot (0.98 % case). In traumatic defects, flaps were done as a primary procedure in 19 (31.15 %) cases after obtaining a healthy milieu after debridement and were done as a secondary procedure in 42 (68.85 %) cases. Cases with flap cover being done as a secondary procedure was higher due to the late referrals, which were treated primarily outside the author’s clinic. However, we prefer doing a primary coverage depending on a satisfactory debridement. A flap was done only after the erythrocyte sedimentation rate and C-reactive protein levels were within normal limits. We did not perform a pre-operative Doppler study or an angiographic study to identify the perforator or the pre-operative vascularity of the flap. A single surgeon performed all the flaps.

This flap was conceived on a sound anatomical basis. The popliteal artery in the popliteal fossa gives two dominant branches, one each to the medial and lateral head of gastrocnemius and another branch, which continues as the sural artery. The sural artery divides into median, lateral and medial branches. The first one is constant and the other two are reciprocal in size. There is also a reciprocal relationship in size between direct cutaneous sural vessels and muscular branches supplying the gastrocnemius heads. The median sural artery accompanies the sural nerve and vein until the proximal calf region. The median sural artery supplies the posterior mid-calf skin and subcutaneous tissue. This suprafascial vascular network arborises both longitudinally and radially and anastomoses with septocutaneous perforators of the peroneal artery in the distal third region of the calf. This reliable plexus between two different sources, the peroneal artery and the median sural artery via the septocutaneous perforators, forms the basis of the distally based fasciocutaneous sural flap from the posterior and posterolateral aspect of the mid-calf region. Among many septocutaneous perfortaors, the distal most is given at 5 cm above the lateral malleolus.

With the patient in the lateral position, the recipient raw area was measured after necessary debridement. The distally based sural flap was marked, over the posterior and posterolateral aspect of mid-calf reaching upto the base of the popliteal fossa in some cases, measuring 2.5 cm more than the recipient area disposed transversely or longitudinally, to cover the defect without tension. The flap base was not distalised beyond 5 cm above the mortise. The length of the flap was need-based but the longest extent in our series went proximally up to the popliteal fossa. Then, the skin and subcutaneous tissue along with deep fascia attached to the skin were dissected and the flap was raised. Tag sutures were taken to twin the skin and the deep fascia. This prevented shearing of the suprafascial vascular plexus. The sural artery, nerve and vein were ligated and cut at the end of the flap near the popliteal fossa. The sural nerve was dissected from the popliteal fossa downwards. Tracking the nerve helps isolate the perforators since they lie along the course of the nerve. The level of the adiposofasciocutaneous pedicle was left as high above the lateral malleolus to enable sufficient pivoting at the pedicle to cover the defect. The flap viability was assessed intra-operatively by deflating the tourniquet. The raised flap was directly rotated and placed over the defect and was loosely sutured to the edges with minimum tension free sutures. No tunnelling was done. An open passage was created for the flap by incising the skin bridge between the donor and recipient area. The flap was not punctured in any of the cases. No drains were used in our series. The donor area was then covered with a split skin graft. The limb was immobilized with a plaster of Paris slab with a non-compressive dressing. The flap was inspected at regular intervals.

## Results

Out of 109 patients operated for the sural flaps, 7 were lost to follow-up. Of the remaining 102 patients, 81 were male and 21 were female. The average age of the patients was 32.7 years (2–65 years). The average size of the flap was 148.10 ± 59.54 cm^2^ (24–308 cm^2^). The average duration of surgery, including preparation of the defect, elevation of the flap, confirming viability, repeat scrubbing and draping for the skin graft donor area and skin grafting, was 121.29 ± 31.16 min, with the average time to raise the flap alone being 34.24 ± 9.34 min. Cases with a defect over the dorsal foot and medial ankle and sole took more time to mobilize and suture the flap.

Of 102 flaps, 91 (89.21 %) cases healed uneventfully. The average healing time was 20.88 ± 6.71 days. There was evidence of distal edge necrosis in nine patients, of which three patients had an edge necrosis of up to 3 cm. All these patients had a soft tissue defect over the dorsum of the foot and ankle. In one patient with a diabetic ulcer over the plantar region of the foot, we lost up to 6 cm of the flap. One flap required flap repositioning since it could not be firmly secured to the raw edges of the soft tissue defect and had slipped. In one patient with fibromatosis, there was a recurrence of the tumor at the flap edges. Though we did have cases with edge necrosis, we did not have any case of postoperative flap infection with seropurulent discharge. Seven (6.86 %) of the patients required repeat grafting at the donor area. Six of these had either a diabetic, venous or leprotic ulcer. A total of 69 cases showed sural hypoaesthesia over the dorsolateral aspect of the foot but none had any functional deficit. A summary of the details of the soft tissue defect and flap is set out in Table 1.

**Table 1:** Patient (*n* = 102) and sural flap details

Characteristic	Number
Age: mean (range)	32.7 years
Sex ratio (M:F)	81:21
Average size of the flap	148.10 ± 59.54 cm^2^

Operation details	No. of cases
Site	
Ankle dorsum	17
Ankle medial	14
Ankle lateral	10
Heel medial	4
Heel lateral	3
Heel rear	30
Heel plantar	23
Foot dorsum	14
Foot plantar	6
Anteromedial mid-1/3 tibia	3
Anterolateral mid-1/3 tibia	0
Anteromedial distal 1/3 tibia	7
Anterolateral distal 1/3 tibia	1
Over tendo-achilles	17
Cause of soft tissue defect	
Trauma	61 (59.80 %)
Diabetic ulcers	12 (11.76 %)
Post-traumatic scar contracture	8 (7.84 %)
Venous ulcer	4 (3.92 %)
Wound dehiscence	10 (9.80 %)
Leprotic non-healing ulcer	1 (0.98 %)
Post-infective wound	1 (0.98 %)
Radiation-induced ulcer following radiotherapy for synovial cell sarcoma	1 (0.98 %)
Post-fibromatosis excision	1 (0.98 %)
Post-dermatofibrosarcoma excision	1 (0.98 %)
Post-heel melanoma excision	1 (0.98 %)
Actinomycosis foot	1 (0.98 %)
Average healing time	20.88 ± 6.71 days
Complications	
Edge necrosis	9
Repeat donor site grafting required	7

## Discussion

There are several techniques to cover the soft tissue defects of distal two-thirds of the tibia and foot [1–13]. The description of the neurocutaneous Masquelet flaps has revolutionised the osteoplastic armamentarium of surgeons not conversant with microvascular free flaps [14]. The reliability of septocutaneous perforators has been well documented. Hence, raising a flap based on this reliable anastomosis of peroneal artery and median sural artery, along with the sural nerve and short saphenous vein has been described to be successful. The posterior and posterolateral aspect of the calf is usually spared in cases of distal lower limb soft tissue defects. In addition, the distally based flap can be easily elevated and rotated to cover the defects over a large area.

The extent of coverage offered by the sural flap extends from the mid- and distal third of the tibia, the posterior and medial aspects of the ankle and heel and the dorsum of foot. The advantage of using this reverse sural fasciocutaneous flap with a cutaneous pedicle is that the skin over the pedicle prevents any chance of torsion of the pedicle which could lead to flap failure. Also, preserving a skin paddle demands using an open bridge technique without tunnelling the skin between the donor area and the wound. This again prevents the chances of pedicle compression due to suturing the bridge over the pedicle as is done in the conventional sural fasciocutaneous flap. There have been reports of using negative pressure wound therapy with drains to decrease the venous congestion in flaps [18]. However, we did not encounter any major flap venous congestion due to the open bridge technique and non-watertight, loose but secure closure with intermittent sutures.

On the other hand, the disadvantage of maintaining the skin over the pedicle while lifting the flap is that it leaves a large donor area defect on the posterior calf that requires a split skin graft. This was unavoidable considering the large defects intended to be covered in this series, which prevented direct skin suturing. Hence, this technique may appear to be cosmetically unappealing as compared to the current technique. This donor defect can be aesthetically covered using full-length trousers. The other disadvantage of using the reverse sural fasciocutaneous flap with a cutaneous pedicle is that it leads to a dog-ear formation after flap rotation at the pivot point. But this dog-ear can be reduced gradually by post-operative compression dressing. We also observed that the more proximal the defect, the less prominent was the dog-ear.

Among the nine cases that had an edge necrosis, most had soft tissue defects over the dorsum of the ankle and foot. The other case with a large flap necrosis had a diabetic ulcer over the plantar aspect of the foot. Thus, we suggest that the chances of edge necrosis of the flap are higher when there is a distal soft tissue defect, i.e. over the dorsum of the ankle and foot and over the sole, more so among patients with diabetic and venous ulcers. All these cases were treated with debridement and split thickness skin grafting. We encountered donor site split skin graft failures in seven patients, most with diabetic, venous or trophic ulcers. This complication must be kept in mind while treating this subset of patients. All these cases, healed with secondary intention after regular dressing. We had three cases with an exposed metallic implant with an ununited underlying fracture. There are reports suggesting a flap cover to be carried out, without an implant removal in spite of presenting 5 months after the index trauma [20]. In our series, too, we preserved the implant and did a flap cover, and all of these healed uneventfully.

There was sural hypoesthesia over the dorsolateral foot, but this does not lead to any functional deficit. This is principally an insensate flap. None of our patients with the flap covering heel defect developed a trophic ulcer. The reason for this observation remains to be investigated. After this flap, the surgeon is prevented from taking any posterolateral bone graft using Harman’s approach in case of tibia delayed union or non-union.

Thus, the reverse sural fasciocutaneous flap with a cutaneous pedicle is a quick, versatile, easy and safe soft tissue defect coverage technique requiring no microvascular repair. However, there is no substitute to careful pre-operative planning of the flap to mobilise and cover the soft tissue defects. We also state the need for a multidisciplinary approach, when indicated, to treat patients with large lower limb soft tissue defects.
